# Systematic review and meta-analysis of public hospital efficiency studies in Gulf region and selected countries in similar settings

**DOI:** 10.1186/s12962-019-0185-4

**Published:** 2019-08-06

**Authors:** Ahmed Alatawi, Sayem Ahmed, Louis Niessen, Jahangir Khan

**Affiliations:** 1 Health Economics Group, Department of Clinical Sciences, Liverpool School of Tropical Medicine, LSTM, Room 1966-215-206, Pembroke Place, Liverpool, L3 5QA UK; 2 Department of Clinical Pharmacy, College of Pharmacy, Al-Jouf University, 2014, King Khaled Road, Sakakah, Saudi Arabia; 3 Health Economics and Financing Research Group, Health System and Population Studies Division, International Centre for Diarrhoeal Disease Research, Bangladesh (icddr,b), Dhaka, Bangladesh; 4 Health Economics and Policy Research Group, Department of Learning, Informatics, Management and Ethics (LIME), Karolinska Institute, Stockholm, Sweden; 5 Division of Health Sciences, University of Warwick, Warwick, UK; 6 Department of International Health Systems, Johns Hopkins Bloomberg School of Public Health, Baltimore, USA

**Keywords:** Gulf countries, Systematic review, Technical efficiency, Public hospitals, Data envelopment analysis, Stochastic frontier analysis

## Abstract

**Background:**

The assessment of hospital efficiency is attracting interest worldwide, particularly in Gulf Cooperation Council (GCC) countries. The objective of this study was to review the literature on public hospital efficiency and synthesise the findings in GCC countries and comparable settings.

**Methods:**

We systematically searched six scientific databases, references and grey literature for studies that measured the efficiency of public hospitals in appropriate countries, and followed PRISMA guidelines to present the results. We summarised the included studies in terms of samples, methods/technologies and findings, then assessed their quality. We meta-analysed the efficiency estimates using Spearman’s rank correlations and logistic regression, to examine the internal validity of the findings.

**Results:**

We identified and meta-analysed 22 of 1128 studies. Four studies were conducted in GCC nations, 18 came from Iran and Turkey. The pooled technical-efficiency (TE) was 0.792 (SE ± 0.03). There were considerable variations in model specification, analysis orientation and variables used in the studies, which influenced efficiency estimates. The studies lacked some elements required in quality appraisal, achieving an average of 73%. Meta-analysis showed negative correlations between sample size and efficiency scores; the odd ratio was 0.081 (CI 0.005: 1.300; P value = 0.07) at 10% risk level. The choice of model orientation was significantly influenced (82%) by the studied countries’ income categories, which was compatible with the strategic plans of these countries.

**Conclusions:**

The studies showed methodological and qualitative deficiencies that limited their credibility. Our review suggested that methodology and assumption choices have a substantial impact on efficiency measurements. Given the GCC countries’ strategic plans and resource allocations, these nations need further efficiency research using high-quality data, different orientations and developed models. This will establish an evidence-based knowledge base appropriate for use in public hospital assessments, policy- and decision-making and the assurance of value for money.

## Introduction

Many nations seek to provide their population with an efficient, equitable and effective healthcare system. This is certainly true of the Gulf Cooperation Council (GCC) countries, which have experienced substantial population growth and increased life expectancy in recent decades. These have, in turn, increased demand for healthcare services [1, 2]. In these countries, average government healthcare spending is 73%, corresponding to 3.2% of GDP in 2013 [3, 4]. Yet while public spending on health is remarkably high in GCC nations, in comparison with many high-income countries, it is rather low as a share of GDP [5]. It has been observed that in Gulf countries, a mere 2.0 hospital beds are allocated per 1000 of population; in contrast, the corresponding figure in other high-income countries is on average 9.0 [6, 7].

Although GCC states spend more than twice as much on health than upper-middle income countries (USD 1100–2000 per capita for GCCs versus USD 505 per capita), the number of hospital beds per 1000 people is fewer, at around 2.0 versus 3.4 hospital beds per 1000 of population [7]. These statistics indicate a potential inefficiency in resource utilization within GCC countries. The healthcare expenditure in GCC nations was expected to rise from USD 55 billion to USD 69.4 billion between 2014 and 2018 [1, 2]. Moreover, demand for healthcare services is expected to increase by 240%, and thus to require many more hospital beds, with a total of almost 162,000 to be provided by 2025 in the GCC [8]. Considering the observed imbalance between health service availability and health spending across countries, better use of resources is fundamental to the achievement of efficiency in health systems [9].

Many national governments worldwide must assess the efficiency of their health sectors, to ensure that public money is used to best effect [10]. A diverse collection of efficiency-related notions and concepts have been used in such efficiency analysis, including theories of technical, allocative, cost and overall efficiency. Of these efficiency concepts, the technical efficiency approach is the most commonly used. It is based on Farrell’s concept that “a hospital that produces the maximum amount of output from a given input, or produces a given output with least quantities of inputs, can be recognised as technically efficient” [11, 12].

Hospital efficiency is crucial for the efficiency of the health system overall, as hospitals are primary consumers of health resources [12, 13]. For instance, Hanson et al. [13] stated, in 2002, that public hospitals consumed a large proportion (around 40%) of the total public health budget in many sub-Saharan African countries. Others have found that public hospitals shared 44% of all national health services’ spending in the United Kingdom in 2012/13 [14].

Globally, the measurement of hospital efficiency has been achieved using various techniques, mainly through frontier analysis methods either as “non-parametric” data envelopment analysis (DEA) or as “parametric” stochastic frontier analysis (SFA). These methods compare hospitals’ actual performance against an estimated efficient frontier, which is deemed to be achieved by the best-performing hospitals [15, 16]. The selection of input and output variables is an essential step in the measurement of such comparative performance, because the results of any efficiency assessment depend significantly on the variables used in the estimation models [17]. To date, the literature has focused on labour (e.g. health professionals) and capital (e.g. number of beds) as the input variables, while few studies have included consumable resources, such as pharmaceuticals [10, 17]. The main categories of output used in efficiency studies comprise healthcare activities, for instance the number of outpatient visits, inpatient services, number of surgeries and health outcomes (e.g. mortality rate) [10].

Despite global interest by researchers and policy-makers, considerable uncertainty exists as to whether the methods frequently applied in efficiency analysis are sufficiently well developed to be useful. There is little consensus regarding the appropriateness of the efficiency measurement and estimation techniques that policy-makers lean on to make decisions about efficient resource allocation [15]. However, while recent decades have seen a growth in research of the supply-side of hospital efficiency, the demand-side (e.g. health policy) remains under-researched [18]. Many in the public health area have maintained a focus on the efficiency of primary health services, neglecting secondary-level hospital services in the process [19]. In general, there is scarcity of scientific studies and empirical works on the efficiency of public hospitals, and such scarcity is particularly pronounced in GCC countries.

To our knowledge, there is no extant systematic review of studies that examines the efficiency of public hospitals in Gulf countries. This study aims to review the existing literature systematically, and to synthesise the findings on public hospital efficiency studies in the GCC region and in countries that are comparable in terms of income level, demographic characteristics and health provision. Specifically, we intend to summarise the included studies regarding their characteristics and capacity to describe health care performance and explain differences in efficiency estimates.

Since exploration of variations in hospital efficiency assessments can yield valuable evidence, we have explored experiences in comparable countries, to enhance our understanding of how efficiency studies have been performed there. Such understanding could helpfully influence policy decisions in the GCC countries. Moreover, we perform a meta-analysis of the efficiency estimates reported in the reviewed studies, to analyse the stability of the efficiency findings.

## Methods

### Search strategy

In July and August 2017, we searched for relevant studies in six indexed scientific databases, namely PUBMED, CINAHL, ECONLIT, MEDLINE, EMBASE and Cochrane, to identify relevant English-language studies indexed at any time. To ensure a broad range of relevant studies, we used an appropriate combination of medical subject heading (MeSH) terms and text words (ti, ab, kw) to search the databases [20]. We also activated the notification alert that registered in the relevant databases for any potential papers that met our search words. The following search algorithm was used: (“efficiency” OR “efficienc*” OR “productiv*” OR “inefficien*” OR “performance” OR “data envelopment analysis” OR “DEA” OR “stochastic frontier” OR “SFA” OR “parametric” OR “non-parametric” OR “nonparametric” OR “healthcare efficiency”) AND (“Hospital*” OR “Public Hospitals” OR “Secondary Care” OR “Public Health Centre” OR “Government* Hospitals”) AND (“High Income” OR “Upper-Middle” OR “Middle Income” OR “Gulf Countr*” OR “GCC” OR “Middle East” OR “Islamic Countries” OR “Single Payer Health System” OR “Saudi Arabia” OR “Iran” OR “Turkey”). The search process complied with PRISMA guidelines [21]. The study protocol was approved by PROSPERO (Protocol ID: CRD42017074582). We identified studies that examined healthcare efficiency measurements and production assessments of public health facilities, both in the GCC countries and in similar settings. All of the studied countries have a high or upper-middle income as defined by the World Bank, a single-payer health system and shared demographic characteristics [22]. We subsequently extended our search by looking through the reference sections of the studies identified in the databases. Moreover, we manually searched the grey literature for potentially relevant articles, because some efficiency measures relevant to GCC states may not have been included in the published literature.

### Inclusion criteria

For a study to be included in the review, it had to satisfy the following inclusion criteria: (1) a study ought to empirically estimate efficiency and report technical efficiency scores. (2) a study must have public hospitals as the unit of analysis. (3) a study must have been conducted in Gulf region (GCC) or similar countries. We excluded studies that failed to empirically assess the efficiency of healthcare centres; for instance, some studies explained efficiency techniques and described methods but did not include empirical data. Studies that focused solely on the private sector were excluded, as were studies that used measures other than efficiency estimates, for example productivity change.

### Region selection

We sought relevant literature that studied GCC countries (Saudi Arabia, United Arab Emirates, Oman, Kuwait, Qatar and Bahrain). We found that Iran and Turkey share relevant characteristics with GCC states, in that both have an upper-middle income, are located in the Middle East and have a public health system funded mainly by the government (i.e. a single-payer system). Like the GCC nations, Iran and Turkey have Islamic cultures and they experience levels and patterns of demand for health activities and services that resemble those of the GCC countries.

### Selection of studies

The author (AA) performed the database search for potential articles, using our search terms and working closely with librarians to refine the search strategy. Two authors (AA and SA) independently screened the titles and the abstracts of all resulting articles, to ascertain whether they met the eligibility criteria and thus reduce the possibility of selection bias. The full texts of all included articles were examined in parallel and separately by the two authors, to determine whether they met all inclusion criteria. Disagreements were resolved by peer discussion, and any differences that could not be resolved were referred to a third member of the review team.

### Data extraction

Two reviewers (AA and SA) performed the data extraction independently. Data extracted for each study comprised: year of publication, number of hospitals included in the study, the studied country, income category of that country, percentage of non-public hospitals in the sample, type of hospital (general and/or specialized), data sources and collection year, estimation methods, input and output variables, technology orientation, model specification, second-stage analysis, sensitivity analysis, and all estimated efficiency scores.

### Quality assessment

We evaluated the quality of the reviewed studies according to four dimensions that were developed by Varabyova and Müller in 2016 [23], based on the quality appraisals of economic evaluations and efficiency measurement studies [24, 25]. These dimensions address reporting, external validity, bias and power. The reporting dimension ensured that the study provides sufficient information to permit a dispassionate evaluation of the outcomes. The external validity element addressed the inclusiveness of the sample. The bias dimension interrogated data accuracy, appropriateness of used techniques, the presence of outliers, and potential bias in the second-stage analysis. The power dimension assessed whether the authors provided evidence to support the study findings [23].

### Meta-analysis

To evaluate the consistency of technical efficiency estimates from different studies, we performed a meta-analysis of the reported findings. For all studies that used panel data and reported a separate score for each year, we calculated the weighted average of these estimates and calculated a pooled technical efficiency (TE) score. The estimated mean of the TE was compared using an independent-samples *T* Test based on different features (such as methods of estimations like DEA, SFA; income levels of the countries) of the included studies. To test the internal validity of the findings, we estimated bivariate Spearman’s rank correlations between efficiency scores and related variables in the reviewed studies, e.g. methods, income levels, number of hospitals. In the logistic regression model, we categorized the TE scores into two levels: ‘0.8 and above’ and ‘less than 0.8′ for use as the dependent variable. Furthermore, we used number of inputs and outputs variables, income-levels of the country (high or upper-middle), number of hospitals, estimation method (DEA or SFA), the orientation of the technology (Input or output), the specification of the model, and quality assessment scores as explanatory variables. We included these characteristics because the literature indicates that heterogeneity across the sample could affect estimated efficiency scores [16]. Data was analysed using IBM SPSS statistic, version 24 as well as STATA version 13.

## Results

**Fig. 1:**
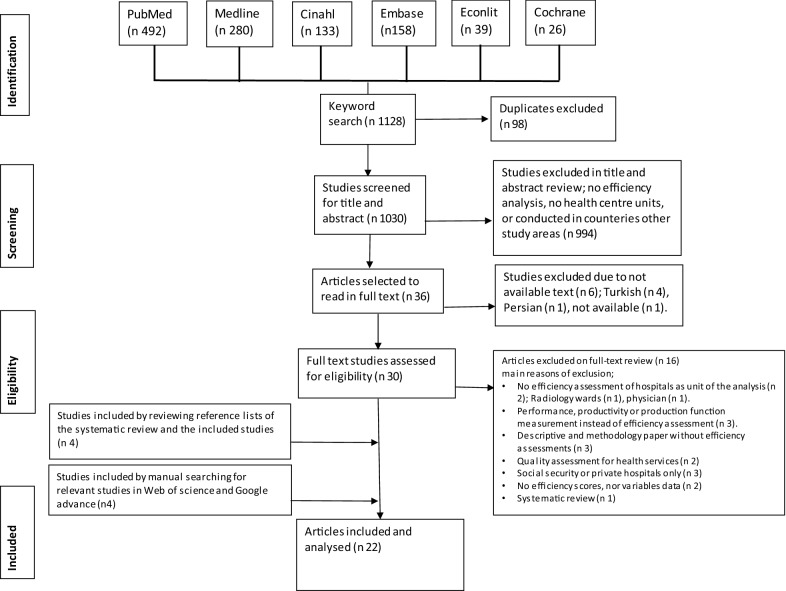
The flow of included studies through phases of the systematic review

Our search of the databases yielded 1128 titles/abstracts. We deleted 98 duplicate records and excluded 994 irrelevant records through title and abstract screening. We also eliminated six records because there was no English-language version available. Thereafter we assessed 30 full-text articles for eligibility and excluded a further 16 because they did not satisfy our inclusion/exclusion criteria. Through reference tracking, we identified four more records and another four publications were identified by manual search of the relevant grey literature. Finally, 22 studies that satisfied our inclusion/exclusion criteria were included in the meta-analysis. Figure 1 summarises the four phases of our systematic literature search following PRISMA guidance.

Table 1 summarises the most prominent characteristics of the 22 studies reviewed. Their publication dates ranged from 2000 to 2017. Of all studies, only four were conducted in high-income Gulf countries: two from Saudi Arabia, one from the United Arab Emirates and one from Oman [28, 45–47]. The remaining 18 studies were conducted in upper-middle income countries: 10 studies were conducted in Iranian hospitals and the remaining eight in Turkish hospitals. The number of sample hospitals per study varied from eight to 1103.

**Table 1:** Summary of the reviewed studies’ characteristics

No.	Study	Publication year	Country	No. of hospitals	Inputs	Outputs	Methods of analysis	Second-stage	Quality (%)
1	Yusefzadeh et al. [26]	2013	Iran	23	Bed, doctor, health personnel	Outpatients, occupied day bed	DEA	NA	75
2	Ahmadkiadaliri et al. [27]	2011	Iran	19	Physician, specialist, nurses and others. bed	Outpatient, inpatients days, surgeries, BOR	DEA	NA	83
3	Helal and Elimam [28]	2017	Saudi Arabia	270	Bed, doctors, nurses, other personnel	Outpatient, inpatients, No radiology, laboratory,	DEA	NA	67
4	Gok and Sezen [29]	2013	Turkey	348	Bed, specialist physicians, non-SP. physicians	BUR, BTR, surgery, births, outpatient inpatient days, discharge.	DEA	Logit regression analysis, correlation, mean difference.	92
5	Gok and Altindag [30]	2015	Turkey	251	Specialized physicians, non-sp. physicians, bed	BUR, BTR, surgery, births, outpatient inpatient days, discharge.	DEA, MPI	correlations, mean differences	92
6	Hatam et al. [31]	2010	Iran	21	Bed, physicians. Nurses, other personnel	BOR, bed, patient admissions, OBD, ALS, BTR	DEA, MPI	NA	62
7	Mehrtak et al. [32]	2014	Iran	18	Bed, physician, nurse, other professionals	Surgeries, discharges, BOR	DEA, Pabon Lasso	NA	77
8	Kalhor et al. [33]	2016	Iran	54	Doctors, nurses, medical personnel, beds.	Patients days, outpatient, surgery, ALS.	DEA	Group comparison	85
9	Rezaee and Karimdadi [34]	2015	Iran	288	Health personals, equipment, bed	Inpatient, outpatient, special patients, bed, BOR	DEA	NA	42
10	Shahhoseini et al. [35]	2011	Iran	12	Physician, nurse, other staff, bed	inpatient days, ALS, BOR, outpatient, operations	DEA	NA	75
11	Ozgen Narci et al. [36]	2015	Turkey	1103	Bed, specialist, general doctor, nurse, and other employees.	Discharges, day-care, surgeries, outpatient, emergency care	DEA	Multivariate Tobit regression	69
12	Sahin and Ozcan [37]	2000	Turkey	80	Bed, specialist, general doctor, nurse, others, revolving expenditure	Outpatient, discharged, hospital mortality rate	DEA	Mean difference	75
13	Sahin et al. [38]	2011	Turkey	352	Bed, physician, nurses, others, operational expenses.	Outpatient, Inpatient, surgeries	DEA, MPI	NA	77
14	Jandaghi et al. [39]	2010	Iran	8	Physicians, nurse, paramedics, administrative, hospital costs	Outpatient, emergency client, bed day	DEA	NA	58
15	Farzianpour et al. [40]	2012	Iran	16	Physicians, nurses, beds	Inpatients, outpatient, ALS	DEA	NA	50
16	Atilgan and Caliskan [41]	2015	Turkey	332	Physician price, ancillary price, administrative price, capital price	Outpatient, inpatient	SFA	Translog cost function specifications. Generalization assessment.	77
17	Atilgan [42]	2016	Turkey	429	Physician, ancillary, administrative staff, bed	Outpatient	SFA	Restricted and unrestricted effect of Cobb–Douglas and Translog model specifications. Correlation	92
18	Atilgan [43]	2016	Turkey	459	Physician, ancillary, administrative staff, bed	Inpatient discharge, patient days	SFA	DISCH and PATDAY model specification, correlations.	85
19	Sheikhzadeh et al. [44]	2012	Iran	11	Specialist physician, general professionals (physician, nurses, residents, medical team), support staff and non-medical teams, bed	Emergency patients, outpatient, inpatient	DEA	Multiple linear regression, correlation	75
20	Mahate et al. [45]	2016	United Arab Emirates	96	Physician, dentist, nurse, midwife, pharmacist, AHP, administrator, other, bed	Inpatient, outpatient, ALS	DEA	Correlation analysis	83
21	Abou El-Seoud [46]	2013	Saudi Arabia	20	Specialist, nurse, allied health, bed	Outpatient, inpatient, laboratory, radiology	DEA	NA	58
22	Ramakrishnan [47]	2005	Oman	20	Bed, doctor, other professionals.	Outpatient, inpatient, major, minor surgical procedures	DEA, MPI	Mean comparison	62

DEA: Data envelopment analysis; BOR: bed occupancy rate; BUR: bed utilization rate; BTR: bed turnover rate; MPI: Malmquist productivity index; OBD: occupancy bed days; ALS: average length of stay; SFA: stochastic frontier saanalysis

Fifteen studies used cross-sectional data, seven used panel data. The health reports, hospital records or annual statistical records were the sources of data in these studies. Regarding methodology, 19 of the 22 reviewed studies used nonparametric methods and the rest applied parametric approaches. Among nonparametric methods, data envelopment analysis (DEA) was predominantly used in 19 studies. Other nonparametric methods included Malmquist Productivity Index (MPI) in four studies [30, 31, 38, 47] and Pabon lasso analysis in one study [32]: both of these methods were used along with the DEA in these cases. Stochastic frontier analysis (SFA) was the exclusive parametric application and used in three studies from Turkish hospitals [41–43]. Efficiency had been assessed in light of various concepts including technical-, scale-, and pure-efficiency with a primary focus on technical efficiency (TE) in the reviewed studies.

The reviewed studies varied in the model specifications they used to estimate the technical efficiency of public hospitals. Among the studies that applied DEA applications, 12 used both constant and variable return to the efficiency scale (CRS and VRS), whereas four studies applied variable return to scale (VRS) and three used constant return to scale (CRS). The three SFA studies used two model specifications in each case to assess efficiency scores, including Cobb–Douglas and translog models. In respect to the orientation of the technology, most (82%) of the studies relied on input orientation, aiming at minimisation of health resources (inputs) for a fixed level of output. In contrast, four studies conducted in GCC countries aimed to enhance the provision of health service by applying output orientation [28, 45–47].

The inputs used in the efficiency analysis of the included studies are presented in Table 1, with a median of four input variables per study with a mean of 3.9 (range: 2–6). Predominant inputs were the capital (number of beds) and labour (number of health workers with different professional categories) variables. Three studies [37–39] used capital expenses in the inputs, and one study [41] included prices of capital and labour. Numerous output dimensions were used in the efficiency models: the mean was 3.7 (range: 1–7) and the median was 3.5 variables. Output variables focused on health care activities and direct patient services. Seven studies used bed turnover (BTR), utilization (BUR) and occupancy (BOR) rates, and five studies used average length of stay (ALS), while one study [37] used mortality rate in its hospitals as output variable.

The last column in Table 1 shows the quality assessment scores of the four dimensions: reporting, external validity, bias, and power. The median quality score was 75% and the mean was 73%; scores ranged from 41 to 92%. The reviewed studies frequently missed points on various dimensions. In the reporting dimension, five studies lacked description of the underlying economic theory and seven studies failed to address the limitations of the study in discussions. In the external validity dimension, the model assumption and appropriateness of the benchmarks was missing in eight studies. In the bias dimension, we found that 14 of the studies (64%) neither addressed nor discussed the potential presence of outliers and data accuracy. In addition, only half of the studies (n = 11) conducted second stage analysis. Nineteen of 22 studies reviewed did not generate confidence intervals for efficiency estimates to reveal statistical power, while just 10 of the studies conducted sensitivity analysis.

Technical efficiency (TE) estimates of the reviewed studies varied from 0.47 to 0.98 with a total average of 0.792, standard error (SE:0.03) (Table 2). The average technical efficiency score was 0.778 (SE: 0.104) in the GCC, where the corresponding score of upper-middle countries was 0.796 (SE: 0.031).

**Table 2:** Technical efficiency (TE) scores

	Mean	Standard error SE	Median	Min	Max
Pooled technical efficiency TE	0.792	0.030	0.828	0.470	0.980
Pure/managerial TE	0.876	0.035	0.935	0.590	0.976
Scale TE	0.892	0.027	0.940	0.670	0.981
Data envelopment analysis DEA	0.791	0.035	0.846	0.470	0.980
Stochastic frontier analysis SFA	0.801	0.036	0.776	0.755	0.871
Upper-middle income	0.796	0.031	0.800	0.557	0.980
High income	0.778	0.104	0.859	0.470	0.923

Moreover, the mean estimate of pure/managerial TE score was 0.875 (SE: 0.035), while scale efficiency was 0.892 (SE:0.027). To examine the consistency of efficiency assessments, we conducted a meta-analysis of the estimated 25 TE scores reported in the reviewed studies.

We estimated Spearman’s rank correlations between TE and predictor variables that included; methods of the analysis, orientation and specification of the models, number of inputs and outputs used, number of hospital in the samples, countries and income categories in the reviewed studies, to test the internal validity of findings. Table 3 illustrates this.

**Table 3:** Spearman’s rank correlation between the efficiency scores and different studies’ characteristics

SSpearman’s rho	Technical efficiency	Number of hospitals	Income categories	Orientation of the model
Technical efficiency				
Correlation coefficient	1.000	− 0.519^**^	0.201	0.279
Sig. (2-tailed)	–	0.008	0.336	0.262
N	25	25	25	25
Number of hospitals				
Correlation coefficient	− 0.519^**^	1.000	− 0.201	− 0.076
Sig. (2-tailed)	0.008	–	0.336	0.765
N	25	25	25	25
Income categories				
Correlation coefficient	0.201	− 0.201	1.000	0.818^**^
Sig. (2-tailed)	0.336	0.336	–	0.000
N	25	25	25	25
Orientation of the model				
Correlation coefficient	0.279	− 0.076	0.818^**^	1.000
Sig. (2-tailed)	0.262	0.765	0.000	–
N	25	25	25	25

Income categories of the studied country (high or upper-middle); orientation of the efficiency model (input or output)

**Correlation is significant at 0.01 level (2-tailed)

We found that the correlations were quite low, and some were even negative. Hospital numbers in the samples were negatively correlated with TE scores, suggesting that models with small sample sizes had provided higher efficiency estimates. Moreover, a logistic regression model (Table 4) confirmed these relationships between the number of hospitals and efficiency scores, with an odd ratio (OR) of 0.081 (95% confidence interval CI 0.005: 1.300; P value = 0.07) at 10% risk level. We also found a significant correlation, of 82%, between countries’ income levels and the orientation of the efficiency model used. Furthermore, studies conducted in high-income countries used output orientation models, which pursued the output-maximisation objective while keeping the inputs constant. The studies performed in upper-middle income countries, in contrast, used input orientation models that aimed to minimise the resources used while keeping output constant.

**Table 4:** Logistic regression between technical efficiency scores and model specifications

Variables	Description	Odds ratio OR (95% coefficient interval)
Methods	SFA (Ref = DEA)	0.700 (0.028;73.113)
Income categories	High income (Ref = Upper middle Income)	3.337 (0.157;70.739)
Number of hospitals	Continuous	0.081* (0.005;1.300)
Number of inputs/outputs	Continuous	0.436 (0.028;6.848)
Constant		4.345 (0.494;38.245)

*P < 0.10

## Discussion

The remarkable growth, in recent decades, of expenditure on healthcare in many countries has directed attention to the analysis of efficiency, the performance of public sectors and the need to provide policy-makers with evidence-based knowledge on which to base informed decisions [5, 48]. We reviewed studies that measured technical efficiency, which is defined by Farrell as producing the maximum amount of output from a specific amount of input or producing a given output from minimum input quantities [11]. We assessed relevant studies conducted in public hospitals in the Gulf, Iran and Turkey. Despite dissimilarities between GCC and Iran and Turkey, there are similarities as well in the culture and the health system. These similarities give the latter two countries justifications to be included in the review and such an inclusion gives the opportunity to share the knowledge across countries in the similar settings for future empirical analyses of the public health systems.

We assessed the impact of model characteristics on the reported efficiency scores using meta-analysis based on 25 extracted observations from 22 different studies. Most of these studies were found in six high-quality databases of scientific publications, but this did not yield studies of GCC countries. We had to search the grey literature for Gulf-focused papers, which were not found in the indexed scientific databases because efficiency analysis is a new approach of research in the Gulf region. The studies found as published literature and those sourced as grey literature were mutually exclusive. To the best of our knowledge, this is the first attempt by researchers to conduct a systematic review and quantify the effect of model specifications on hospital efficiency scores in the GCC countries and comparable nations.

We found that DEA was the dominant method by which public hospital efficiency was assessed in the reviewed studies: just three studies applied the SFA method, all conducted in Turkey [41–43]. In the Gulf region and in Iran, efficiency was exclusively measured via DEA and other systematic reviews have found the same method to be common internationally [12, 25]. The use of DEA is well justified by its capability to handle multiple inputs and outputs in different units, and also its functional flexibility in practical application [10, 49].

The reviewed studies originating from Iran and Turkey primarily used the technology orientation of input, whereby output was fixed, and the scholars explored proportional reduction in the input. Such an approach is very practical, since hospital managers and policymakers have more control over inputs than they have over outputs, as shown in previous research [50, 51]. In contrast, two of the four studies arising from Gulf countries applied an output orientation model [45, 47], while the remaining two studies employed both input and output orientation model [28, 46]. Thus, the health-related policy objective within the GCC was to retain the inputs and explore proportional expansion in output. This approach complements the target of Gulf governments, which is to enhance the provision of national and domestic health services to meet the growing demand for healthcare. In such countries, this is the primary goal of health care development strategy plans [2, 52]. Furthermore, this approach was appropriate because reduction of the existing health resources is not the priority of Gulf nations’ health strategies, at least in recent years [2, 45].

Our meta-analysis showed no significant differences between the estimated efficiency in both technology orientations of efficiency analysis. Due to the scarcity of efficiency estimates and related knowledge in the Gulf region, we encourage further investigation and more research in this area. Ideally such study should be undertaken using a variety of technology orientations, considering the goals and functions of the public hospitals.

The studies we reviewed often had limitations, which included aggregation of inputs, mainly in the labour category [27] and aggregation of costs of different types of capital and labour prices [41]. Outputs mainly focused on healthcare activities, ignoring health outcomes and offering no adjustment for differences in case mix or quality of care across hospitals. This might be the reason for high efficiency scores in some hospitals, despite a low quality of care [51]. Further limitations were heterogeneity in sample (number and size of hospitals in each study; activities of the hospitals, etc.), which might affect efficiency scores since in general, the studies did not make appropriate adjustments in light of such heterogeneity. The studies often failed to describe the causes of inefficiency, did not try to evaluate the misspecification in efficiency models and also lacked internal validity of efficiency findings, which could skew the policy implications. Moreover, like Varabyova in 2016, we found that the quality assessment of the studies revealed frequent failure to report production theory and the absence of justification/rationalisation of model assumption choices, reporting study limitations and the presence of outliers [23]. These limitations raised many issues of accuracy, reliability and generalizability of these studies. We suggest that researchers concentrate on the characteristics of the efficiency models and related methodological issues, and encourage transparent reporting of the relevant findings.

We observed, as other authors have done, that scarcity of data underlies many of these limitations. Most studies included in this review selected their variables according to the available secondary data sources, rather than collecting new and more relevant data to construct the best possible measure of performance [51, 53]. It has been argued (separately) by Afzali [17] and Hollingsworth [12] that many hospital databases suffer from insufficient data regarding a broad range of hospital functions and quality of care, including preventive care, health promotion and staff development activities. The GCC Health report 2015 confirms that the same data discrepancies occur in the GCC [2]. Thus, improving hospitals’ databases, through quality data collection and processing techniques, the inclusion of data from different health provision levels, and the capture of valid data that reflects the demand, quality of care and pattern of activities around health care are critical steps towards better quality hospital efficiency studies [17, 53]. Such improvements would enhance further efficiency research by indicating the weaknesses in healthcare production process, and as a result would guide the policy-decision makers to potential reforms in the region.

The findings from our meta-analysis showed no significant differences in the estimated efficiency scores, irrespective of the analysis methods employed, i.e. SFA and DEA. Among the Turkish papers, three studies applied SFA methods and five used DEA. Although SFA reported higher efficiency scores, the difference was not statistically significant and such finding was along the same lines as most previous reviews [12, 50].

Technically, in the DEA approach the entire distance from a decision-making Unit (DMU) to the efficient frontier measures the inefficiency, while in SFA this distance includes both inefficiency and estimation error and consequently, the inefficiency shows a higher value in DEA than in SFA even if we use the same data [54]. Although the choice of DEA or SFA may have a substantial impact on the results, there is no agreement in the literature as to which of these methods reflects the best practice [10, 25]. However, the choice of nonparametric and/or parametric methods in any analysis relies on the specification of the production function, the assumptions about the distribution of the error components, production theory orientations and the perspective of selecting returns to scale assumptions [23, 25]. Our analysis in this study found that DEA studies that applied VRS reported higher efficiency scores, though not to a significant extent, compared with those which used CRS assumptions, since the DEA under VRS assumption tightly enveloped the data and more hospitals were placed on the frontier [10, 25].

Our analysis found a negative relationship between sample size and the estimated efficiency scores, as observed in other studies [36, 40]. Similar findings have been reported in previous literature reviews, which argued that inflated efficiency scores may occur with small sample size due to sparsity problems, meaning that a hospital can be considered efficient just because there is no comparator within the sample [12, 16, 25]. Moreover, overestimates of efficiency scores on DEA can occur if the number of hospitals is small relative to the number of input and output variables [49]. Several empirical analyses have had a small sample size in comparison with the number of the variables used and reported high-efficiency scores [27, 31, 35, 39, 40]. To remedy such problems, Hollingsworth suggested that the number of units used in efficiency assessment should be at least three times the combined counts of inputs and outputs altogether [49]. Apparently, further development of the efficiency models to meet the complexity of production in the public hospitals and demonstration of the efficiency findings is required.

Although we conducted a comprehensive literature search across several databases in our current review, we might have missed some relevant studies. To overcome this, we hand-searched the references and the grey literature to identify more studies. Our findings regarding SFA could be better justified if more than three studies had been found for critical analysis in this review. The study site chosen for our review (the Gulf region), however, may generate strong interest among policy-makers, stakeholders, researchers and academics. Another interesting point arising from our review of studies of Gulf Region is that the output-orientation was mostly preferred to the input-orientation, while studies originating in other countries commonly used the input-orientation.

## Conclusions and recommendations

This systematic review, the first of its kind to focus on the Gulf region, is expected to contribute to the body of knowledge and efficiency studies that my be used to plan future research and policy in the region. Our review has suggested that the methodology choices and technology assumptions exert a high degree of influence on efficiency assessments, as has been found in literature reviews globally.

The number of studies conducted in the Gulf region was remarkably limited and the quality of those reviewed studies was poor in comparison with other relevant studies from other countries. The data used in the reviewed studies had considerable deficiencies for performing high quality efficiency estimates. The Gulf country studies focused on the output-orientation, unlike the reviewed studies in other countries which considered input-orientation. Estimations should, however, take the resource allocation policy in public hospitals into account while planning any efficiency analysis.

Our recommendations could be useful to researchers and policy-makers. In order to create evidence-based scientific knowledge for policy-building, studies of public hospital efficiency should develop compatible high-quality data: this should cover all health care activities and services, and their health outcomes. Public hospital efficiency analyses, which are currently rare in the Gulf region, should be conducted on a much larger scale in order to create more, and validated, knowledge for use in policy-making. Such new studies should employ different methodologies, and assumptions and sensitivity analyses, to validate the findings around public hospital efficiency. Considering the strategic plans and goals of the governments about resource allocations and value for money in public hospitals, future researchers should make the base-case in their analyses.

Finally, to make the best practical use of such research in relation to policy and practice, relevant stakeholders should utilize the knowledge arising from efficiency studies in the Gulf region to convince their policy-makers to develop or amend policies in accordance with national requirements.

## Data Availability

Details of the review protocol and full search strategy are available on PROSPERO (http://www.crd.york.ac.uk/PROSPERO; registration number CRD42017074582). Further data and materials can be requested from the authors.
