# Correction to: Systematic review and meta-analysis of public hospital efficiency studies in Gulf region and selected countries in similar settings

**DOI:** 10.1186/s12962-020-0201-8

**Published:** 2020-02-07

**Authors:** Ahmed D. Alatawi, Sayem Ahmed, Louis Niessen, Jahangir Khan

**Affiliations:** 1 Health Economics Group, Department of Clinical Sciences, Liverpool School of Tropical Medicine, LSTM, Room 1966-215-206, Pembroke Place, Liverpool, L3 5QA UK; 2 Department of Clinical Pharmacy, College of Pharmacy, Al-Jouf University, 2014, King Khaled Road, Sakakah, Saudi Arabia; 3 Health Economics and Financing Research Group, Health System and Population Studies Division, International Centre for Diarrhoeal Disease Research, Bangladesh (icddr,b), Dhaka, Bangladesh; 4 Health Economics and Policy Research Group, Department of Learning, Informatics, Management and Ethics (LIME), Karolinska Institute, Stockholm, Sweden; 5 Division of Health Sciences, University of Warwick, Warwick, UK; 6 Department of International Health Systems, Johns Hopkins Bloomberg School of Public Health, Baltimore, USA

## Correction to: Cost Eff Resour Alloc (2019) 17:17 10.1186/s12962-019-0185-4

Please note that following publication of the original article [1], two errors have been flagged by the authors.

Firstly, the article has been processed with the wrong article type: it is not a ‘Review’, but rather a ‘Research article’.

Secondly, the initial of the corresponding author’s middle name is missing in the original article; please see the corrected name in the author list of this Correction.
